# A Practical Guide to Optimal Adaptive Two‐Stage Designs for Various Endpoint Types

**DOI:** 10.1002/pst.70129

**Published:** 2026-09-29

**Authors:** Nico Bruder, Jan Meis, Maximilian Pilz, Meinhard Kieser

**Affiliations:** ^1^ Institute of Medical Biometry University of Heidelberg Heidelberg Germany; ^2^ Institute of Medical Statistics Medical University of Vienna Vienna Austria; ^3^ Nürnberg School of Health Ohm University of Applied Sciences Nuremberg Nuremberg Germany

**Keywords:** adaptive design, clinical trial, interim analysis, optimal design, sample size determination, statistical hypothesis testing

## Abstract

Adaptive designs are frequently used to adjust sample sizes based on interim data, ensuring reliable conclusions while protecting resources. To maximize trial efficiency, design parameters can be optimized to minimize an objective function (e.g., the expected sample size) while rigorously controlling overall error rates or further operating characteristics. However, existing methodological guidance for optimal adaptive designs mainly focuses on normally distributed data or discrete optimizations for binary data, leaving a limited practical framework to practitioners who work with other common endpoint types. We address this by demonstrating how the asymptotic normal approximations of standard test statistics enable the optimization framework to be systematically applied across diverse endpoint types, including binary and time‐to‐event data. Furthermore, we highlight critical pitfalls in using optimal adaptive designs for two‐sided testing. The results demonstrate that optimizing design parameters can substantially decrease expected sample sizes and improve conditional power compared to standard group‐sequential designs. Using this guidance, we aim to enable researchers to efficiently plan clinical trials in a broad spectrum of clinical settings, a factor that is especially crucial in rare disease research where only limited patient populations are available.

## 1 Introduction

Adaptive designs are a valuable option to achieve the goal of an adequate sample size of a clinical trial. The adaptation of the design at one or several interim analyses makes the design more robust against uncertain planning assumptions. To ensure the protection of the overall Types I and II error rates, it is recommended to define the entire adaptation procedure already during the planning stage [1]. In this case, one may strive for choosing the best possible, that is, the *optimal*, adaptive design.

The task of optimizing adaptive designs has been the topic of extensive research. A popular optimal group‐sequential design for single‐arm trials with binary outcome was proposed by Simon [2]. Further work on discrete optimization is provided, among others, in [3, 4, 5]. Building on the pioneering work of Schmitz [6], general solution strategies to optimizing adaptive designs for normally distributed test statistics were presented in [7, 8, 9]. Open‐source software is provided by Kunzmann et al. [10] and applied by Pilz et al. [11] to explore different optimality criteria and constraints in the case of a z‐test. Furthermore, various work exists on optimizing only particular elements of adaptive designs. Jennison and Turnbull [12] showed how to choose an optimal sample size function for a given conditional error function. Brannath and Bauer [13] and Pilz and Kieser [14] analyzed optimal conditional error functions. Since non‐monotonic conditional error functions may not be desirable, Brannath et al. [15] presented optimal monotonic conditional error functions. A comprehensible overview of the existing literature on optimal adaptive designs and on group‐sequential/adaptive methods in general can be found in the book by Wassmer and Brannath [16].

Optimizing adaptive designs might be of particular interest for rare disease studies. In these trials, the potential number of patients is limited, hence optimizing the (expected) sample size of a design while maintaining a certain power level might be a crucial factor for the feasibility of a trial. When sample sizes are so small that the normal approximation of the test statistic fails, Kieser et al. [17] present results when using a t‐distribution instead of the normal approximation for continuous data.

While broad research on optimal adaptive two‐stage designs exists, only little guidance is given on how to act with non‐normal endpoints. Moreover, the previously mentioned publications on discrete optimization specifically cover binary data. However, discrete optimization is computationally expensive for larger sample sizes, and a continuous approximation is based on the central limit theorem in these cases. In this manuscript, we provide guidance on extending the optimization framework to diverse endpoint types by using their respective asymptotic normal test statistics. The paper offers a practical guide to selecting an optimal adaptive two‐stage design, demonstrates its application across multiple scenarios, and explores caveats when extending the theory to more than two trial groups or two‐sided testing problems.

Throughout this manuscript, we focus on frequentist confirmatory two‐stage adaptive trials with rigorous control of the Type I error rate (or family‐wise error rate). Non‐confirmatory exploratory settings and alternative frameworks like Bayesian adaptive designs or decision‐theoretic designs fall outside the scope of this work.

The manuscript is structured as follows: In Section 2, we present the general solution strategy and explore this strategy for different endpoint distributions in the following sections. These sections cover two‐stage designs with two treatment groups and normally distributed endpoints (Section 3), binary endpoints (Section 4), and time‐to‐event data (Section 5). They always start with a short methodological recap of the respective test statistics and their distributions and follow with a real‐world example illustrating the computation of an optimal adaptive design. Section 6 is dedicated to new insights into the case of two‐sided testing, which exhibit some pitfalls in the usage of optimal adaptive designs. The following Section 7 discusses the extension of the optimization framework to more than two groups. We conclude with a discussion in Section 8.

## 2 Optimizing Adaptive Two‐Stage Designs

### 2.1 Background

Let θ denote our parameter of interest. We want to test a null hypothesis $H_{0}:\theta \leq \theta_{0}$ against an alternative hypothesis $H_{1}:\theta >\theta_{0}$ (or the two‐sided versions of these hypotheses) in a two‐stage adaptive design. The designs presented in this manuscript can be represented as a five‐tuple.

$$D≔\left(n_{1},c_{f},c_{e},n_{2}\left(\cdot \right),c_{2}\left(\cdot \right)\right).$$

The parameter $n_{1}$ denotes the group‐wise sample size in the first stage. The parameters $c_{f}$ and $c_{e}$ are the critical values for the first‐stage test statistic. This means that we compute a test statistic $X_{1}$ at the interim analysis, and if $X_{1}<c_{f}$, we stop the trial for futility (i.e., with acceptance of the null hypothesis), and if $X_{1}>c_{e}$, we stop for efficacy (i.e., with rejection of the null hypothesis). If $X_{1}\in \left[c_{f},c_{e}\right]$, we continue to the second stage. The function $n_{2}\left(\cdot \right)$ defines the group‐wise sample size in the second stage as a function of the first‐stage test statistic $X_{1}$. The function $c_{2}\left(\cdot \right)$ defines the critical value for the second‐stage test statistic $X_{2}$ as a function of $X_{1}$. At the final analysis, we compute $X_{2}$ from the $2n_{2}\left(X_{1}\right)$ patients and reject the null hypothesis if $X_{2}>c_{2}\left(X_{1}\right)$.

In the rest of this manuscript, the second‐stage test statistic without a star (i.e., $X_{2}$) always refers to the second‐stage‐only data, while the second‐stage test statistic with a star (i.e., $X_{2}^{*}$) refers to the statistic computed from the cumulative data of both stages. Because $X_{2}$ is defined to be calculated from the second stage only, the distributions of $X_{1}$ and $X_{2}|X_{1}$ are independent by construction. If the test statistics $X_{1}$ and $X_{2}$ (approximately) follow a normal distribution $N\left(\sqrt{n}\theta ,\sigma^{2}\right)$ and no sample size adaptations are made, it is easy to show that:

(1) $$X_{2}^{*}\overset{d}{=}\sqrt{\frac{n_{1}}{n_{1}+n_{2}}}X_{1}+\sqrt{\frac{n_{2}}{n_{1}+n_{2}}}X_{2}.$$

How to choose the values of the five‐tuple D is not clear beforehand, but one usually imposes restrictions on the design's Types I and II error rates. A common choice is using a constant value for $n_{2}$ and a constant value as the critical value for $X_{2}^{*}$. Such a design is commonly known as group‐sequential design. In the literature, there are two frequently used variants of group‐sequential designs. The first one uses critical values going back to O'Brien and Fleming [18], where $c_{f}=-\infty$ and the parameter c is chosen such that:

$$P_{H_{0}}\left(X_{1}>c\right)+P_{H_{0}}\left(X_{2}^{*}>c/\sqrt{2},X_{1}<c\right)=\alpha .$$

The second popular group‐sequential design was introduced by Pocock [19]. Here, $c_{f}=-\infty$ and the parameter c is chosen such that:

$$P_{H_{0}}\left(X_{1}>c\right)+P_{H_{0}}\left(X_{2}^{*}>c,X_{1}<c\right)=\alpha .$$

For two stages, we can numerically compute that for α=0.025, the O'Brien‐Fleming design has boundaries $c_{e}=2.796$ and $c_{2,*}=1.977$. Using the relationship in Equation 1, we can derive the critical value for the second‐stage‐only test statistic as $c_{2}\left(X_{1}\right)=\sqrt{2}\times 1.977-X_{1}$. The Pocock design has boundaries $c_{e}=c_{2,*}=2.178$ and thus $c_{2}\left(X_{1}\right)=\sqrt{2}\times 2.178-X_{1}$.

Of course, the general representation as five‐tuples allows for far more flexible designs. In particular, the sample size might be adapted at the interim analysis depending on how promising the results are. One way to adapt the sample is based on conditional power [20]. The conditional power is defined as the probability to reject the null hypothesis at the end of the trial given the data observed at the interim analysis, so:

$${\mathrm{CP}}_{\theta }=P_{\theta }\left(X_{2}>c_{2}\left(x_{1}\right)|X_{1}=x_{1}\right).$$

Whether to choose θ as the effect size under the alternative hypothesis or the observed effect size at the interim analysis is a matter of debate, see [21]. The second‐stage sample size can then be adapted such that ${\mathrm{CP}}_{\theta }$ does not fall below a defined threshold, no matter the observed outcome at the interim analysis.

Another way to adapt the sample size was proposed by Metha and Pocock [22], where they introduced the so‐called promising zone design. Their approach also uses the conditional power at the interim analysis, but instead of ensuring a minimum conditional power, they define a range for the conditional power at the interim analysis, the so‐called promising zone. If the conditional power falls into this zone, the sample size is increased to achieve a target conditional power, up to a pre‐defined maximum allowed sample size. If the conditional power is below the promising zone, the trial is either stopped for futility or it continues to Stage 2 without any adaptations, and if it is above the promising zone, the trial continues with the originally planned sample size.

### 2.2 Optimization Procedure

In the framework introduced in [9], the five‐tuple D is chosen via a constrained optimization procedure. The objective function to be optimized can be freely specified. This allows to take a wide range of design goals and trade‐offs into account, depending on what design characteristics are preferable in each specific scenario. As the number of patients is often a restricted resource, in particular in rare disease trials, a common goal is the minimization of the expected sample size ${\mathrm{ESS}}_{\theta }=E_{\theta }\left[n_{1}+n_{2}\left(X_{1}\right)\right]$ of the trial. How to choose the parameter θ for the expectation might be decided on a case‐by‐case basis. One might, for example, choose θ as the effect size under the null hypothesis, the alternative hypothesis, or a (weighted) combination of them. In the following sections, we will often use the latter approach, so the objective function is given as:

$$\bar{\mathrm{ESS}}=\frac{1}{2}{\mathrm{ESS}}_{H_{0}}+\frac{1}{2}{\mathrm{ESS}}_{H_{1}}.$$

It is clear that the optimization must be constrained by defining key requirements our design should fulfill. Naturally, these could include the Type I error rate and the Type II error rate. However, other constraints are imaginable as well. For example, we might want to restrict the maximum sample size [11] or require a minimum conditional power to avoid a second stage without any prospect of success.

In summary, given an objective function g, the optimal adaptive two‐stage design is found as the solution of the following optimization problem:

$$\begin{array}{ll} D_{\mathrm{opt}} & =\underset{D}{\text{argmin}}\;g\left(D\right)\\ & \;\text{subject to}:\;\text{type}\;I\;\text{error rate}\left(D\right)\leq \alpha \\ & \;\text{power}\left(D\right)\geq 1-\beta \\ & \;\vdots \end{array}$$

In order to be able to evaluate the objective function and the constraints for a given design D, it is important that these quantities only depend on the probability distribution of the test statistics $X_{1}$ and $X_{2}$ under specified values of θ. For example, denoting their respective cumulative distribution functions with $F_{i}^{\left(\theta \right)}$ and the densities with $f_{i}^{\left(\theta \right)}$ (i=1,2) for a given value for θ, the Type I error rate and the power of our design can be calculated via:

$$\begin{array}{ll} & P_{\theta }\left(X_{1}>c_{e}\right)+P_{\theta }\left(X_{2}>c_{2}\left(X_{1}\right)\cap X_{1}\in \left[c_{f},c_{e}\right]\right)\\ & \;=1-F_{1}^{\left(\theta \right)}\left(c_{e}\right)+\int_{c_{f}}^{c_{e}}\left(1-F_{2}^{\left(\theta \right)}\left(c_{2}\left(x_{1}\right)\right)\right)f_{1}^{\left(\theta \right)}\left(x_{1}\right){\mathrm{dx}}_{1}, \end{array}$$

where $F_{i}^{\left(\theta \right)}$ and $f_{i}^{\left(\theta \right)}$ depend on the value of θ under the respective hypothesis. Other quantities like the expected sample size can be expressed analogously.

For the optimization itself, we have the problem that the parameter‐space of D is infinite‐dimensional, as $n_{2}\left(\cdot \right)$ and $c_{2}\left(\cdot \right)$ are functions. Kunzmann et al. [10] therefore discretize the parameter space by using a finite number of pivots $x_{1},\ldots ,x_{l}\in \left[c_{f},c_{e}\right]$ for the functions $n_{2}\left(\cdot \right)$ and $c_{2}\left(\cdot \right)$, and afterwards interpolate the functions for the values between the pivots.

Overall, the general strategy to select D can be summarized in a five‐step procedure, see Figure 1.

**FIGURE 1:**
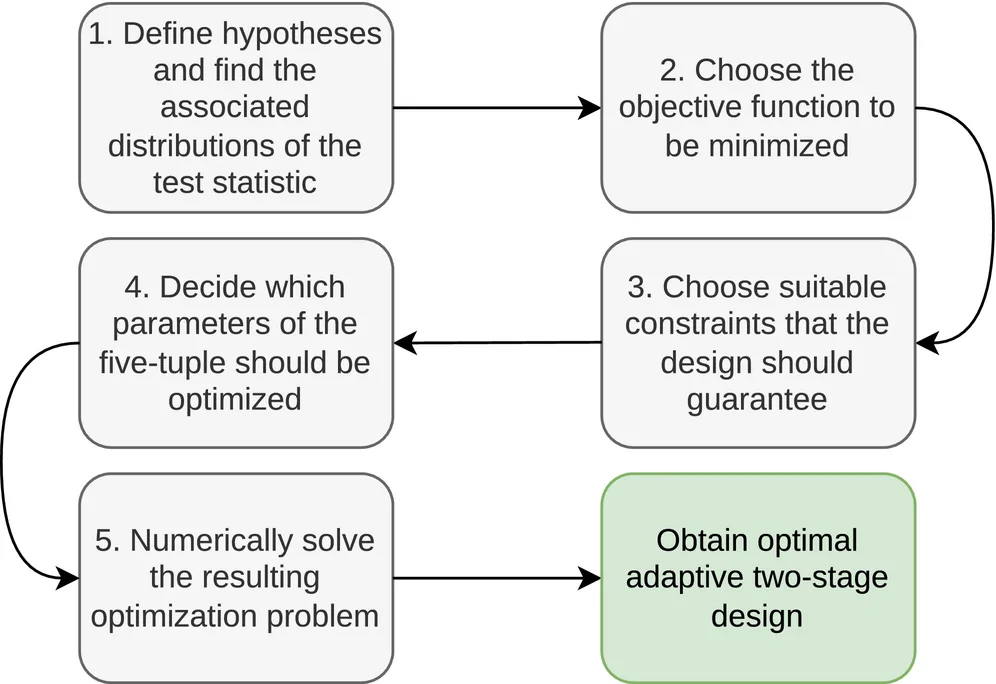
Visualization of the five‐step procedure to determine an optimal adaptive design.

In the following sections, we will explore this general strategy for different types of endpoints and associated test statistic distributions by means of real clinical trials. All examples are computed using the statistical software R [23] and the package adoptr [10]. The source code is available as Supporting Information.

## 3 Normally Distributed Endpoints

### 3.1 Methodology

For normally distributed endpoints and a two‐group comparison, we can compute a Z statistic when the variance in the arms is known. This scenario perfectly fits the optimization framework described in Section 2.2. When writing $\delta ≔\left(\mu_{E}-\mu_{C}\right)/\sigma$ for the standardized treatment effect size between the experimental group and the control group, the hypotheses are given by:

$$H_{0}:\delta \leq 0,\;H_{1}:\delta >0.$$

The stage‐wise test statistics then follow a normal distribution $Z_{i}∼N\left(\sqrt{\frac{n_{i}}{2}}\delta ,1\right)$, where i∈{1,2}.

As a result, we know the distribution of the test statistics under both the null and the alternative. Therefore, we can compute the objective function and the constraints for any given design D, and select the five tuple that minimizes the objective function while fulfilling the constraints.

It is important to note that this formulation assumes a known variance. In practice, the standard deviation of the outcomes is rarely known a priori, and the t‐test is applied by replacing the standard deviation in the test statistic with its empirical estimate. In this case, for each value of δ, the test statistic at stage i follows a non‐central t‐distribution with $2n_{i}-2$ degrees of freedom and non‐centrality parameter $\sqrt{\frac{n_{i}}{2}}\delta$. This exact distribution can then be used for the computation of optimal adaptive two‐stage designs in the exact same way as the normal approximation. Because the normal approximation has smaller tails than the t‐distribution, sample sizes are slightly increased when using t‐distributions to maintain strictly controlled error rates. Acknowledging this distinction is particularly relevant in rare disease research, where sample sizes are typically small to moderate and asymptotic approximations are more likely to fail. A broader description of this issue, accompanied by examples, is provided in [17].

### 3.2 Example

In the BrainSTEADy trial [24], Paret et al. investigate whether fMRI‐neurofeedback is superior compared to sham‐control feedback in treating Borderline‐Personality Disorder. This is a randomized, controlled trial with equal allocation and conducted with an adaptive design. The primary endpoint is captured by the statement “At the moment I don't feel well” on a visual analogue scale (0: “no” −100: “very much”). As the question is asked multiple times, the primary endpoint is analyzed using a mixed linear model. For illustrative purposes, we assume one baseline measurement and one measurement at the end of the intervention here. In a pilot trial, a mean improvement in the score of 0.37 in the treatment group and a mean improvement of 0.03 in the control group were observed with a joint standard deviation of 0.46. This results in an effect size of ${\hat{\delta }}_{\text{pilot}}=0.74$.

In this paper, for power calculations, a clinically relevant effect size of $\delta_{1}=0.5$ is used and the standard deviation of 0.46 is assumed to be known. The original trial design was as follows: In the first stage, $n_{1}=41$ patients were recruited. The first‐stage test statistic is evaluated according to the rule of Bauer and Köhne [25]. Thus, the trial is stopped for futility if $X_{1}<0.52$ (corresponding to a *p*‐value of 0.3) and for efficacy if $X_{1}>2.22$ (corresponding to a *p*‐value of 0.0131). In case of entering the second stage, another batch of $n_{2}=41$ patients was to be recruited. The design is shown in Figure 2 (dashed line). Under our simplified assumption of normal distribution, it shows a power of 85.9% to detect the effect size of $\delta_{1}=0.5$ and a minimal ${\mathrm{CP}}_{\delta =0.5}$ of 51.1% for this effect size in case of entering Stage 2.

**FIGURE 2:**
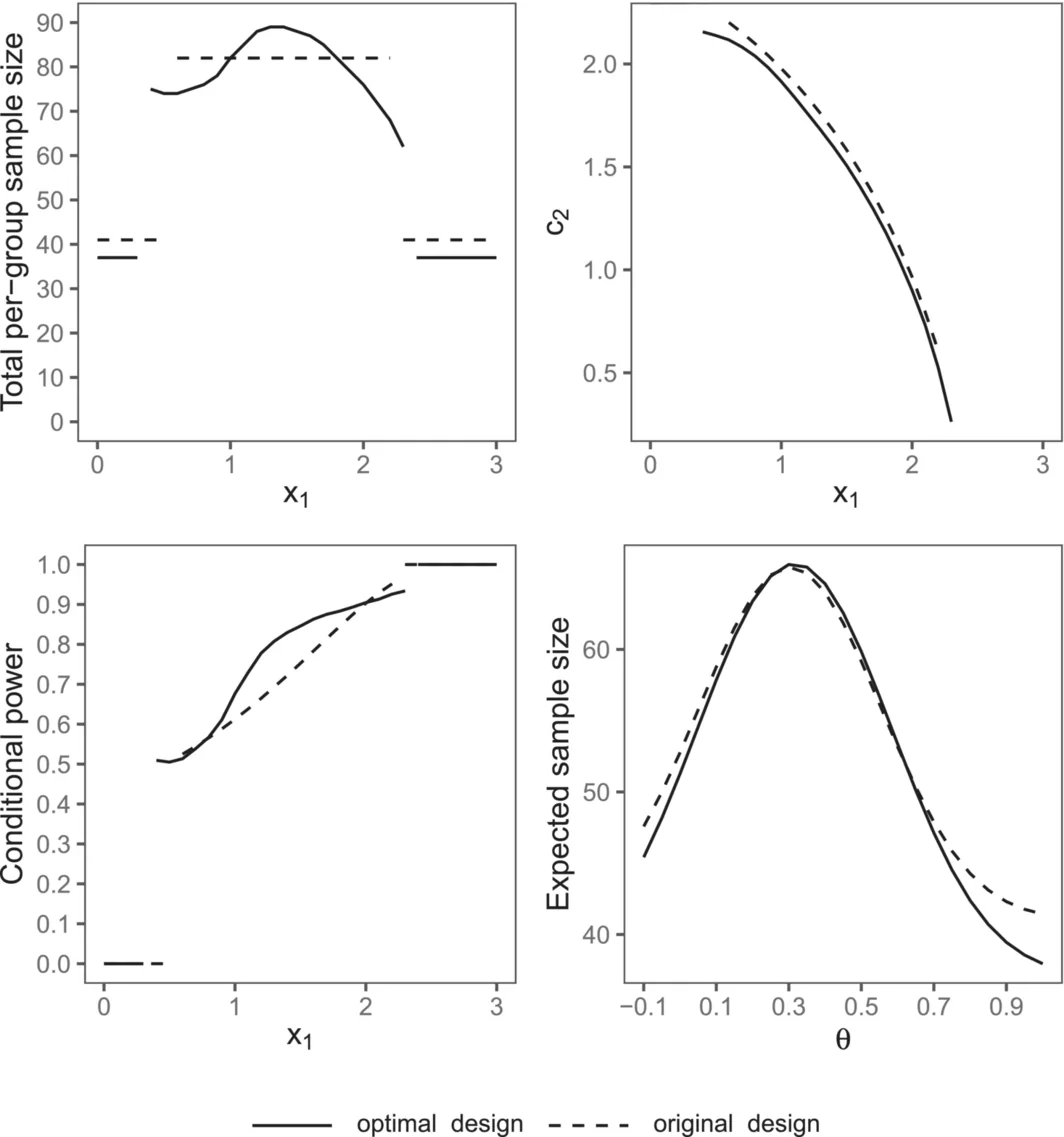
Comparison of original group‐sequential design and optimized adaptive design for the BrainSTEADy trial.

While there are good reasons to use a group‐sequential design (restricted maximum sample size, statistical simplicity), we illustrate its differences to an optimal adaptive design whose primary operating characteristics are close to the original design. We follow the strategy we outlined in Section 2.2:

1. Test statistic and hypotheses are formulated according to Section 3.1.
2. The objective function is chosen as the average of the expected sample size under the null and the alternative hypothesis.
3. The design should show the same Type I error rate (α≤0.025), power (1−β≥0.859), and minimal ${\mathrm{CP}}_{\delta =0.5}$ (${\mathrm{CP}}_{\delta =0.5}\geq 0.511$) as the original design. In addition, the risk to mistakenly stop for futility although the alternative hypothesis is true should not be higher than in the original design (4.1%).
4. We optimize the entire design, that is, all the five parameters of the five‐tuple.
5. We solve the resulting optimization problem.

The resulting optimal design is shown in Figure 2 (solid line). We observe small efficiency gains from an average expected sample size of 55.96 in the original design to 55.56 in the optimal design. This comes with the price of an increased maximum sample size from 82 (original) to 89 (optimal). The optimal design has a first‐stage sample size of 37 and a slightly wider continuation region, and the CP is slightly increased.

To decrease the maximum sample size, it is possible to include this score in the objective function. The objective function then might look like:

$$g\left(D\right)=w_{1}{\mathrm{ESS}}_{H_{0}}+w_{2}{\mathrm{ESS}}_{H_{1}}+w_{3}n_{\max}.$$

In conclusion, we see that the original design is already close to the optimal design, and thus the efficiency gain is small in this case. Hence, the optimal design is not always substantially better than conventional approaches.

In this example, we have assumed that the variance is known. If we would have used the t‐distribution instead, the sample size and the critical values would have slightly increased to maintain the exact error rates (approximately one patient per group). In the Supporting Information, you can find an example of how to compute the optimal design using the t‐distribution.

## 4 Binary Endpoints

### 4.1 Methodology

In the case of binary endpoints, we typically compare the probability $p_{E}$ of an event in the experimental group with the probability of an event $p_{C}$ in the control group. Without loss of generality, we assume that smaller values of $p_{E}$ are favourable and thus the hypotheses are:

$$H_{0}:p_{C}\leq p_{E},\;H_{1}:p_{C}>p_{E}.$$

Under the assumption of a sufficiently large sample size, a suitable test statistic is based on the normal approximation of the rate difference, given by:

$$U_{i}=\sqrt{n_{i}}\frac{{\hat{p}}_{C}^{\left(i\right)}-{\hat{p}}_{E}^{\left(i\right)}}{\sqrt{2{\hat{p}}_{0}^{\left(i\right)}\left(1-{\hat{p}}_{0}^{\left(i\right)}\right)}},\;i\in \left\{1,2\right\}.$$

here ${\hat{p}}_{E}^{\left(i\right)}$ and ${\hat{p}}_{C}^{\left(i\right)}$ denote the empirical rates, and ${\hat{p}}_{0}^{\left(i\right)}=\left({\hat{p}}_{E}^{\left(i\right)}+{\hat{p}}_{C}^{\left(i\right)}\right)/2$ is the pooled rate estimate. Define $p_{0}=\left(p_{E}+p_{C}\right)/2$, $\sigma_{0}=\sqrt{2p_{0}\left(1-p_{0}\right)}$ and $\sigma_{A}=\sqrt{p_{E}\left(1-p_{E}\right)+p_{C}\left(1-p_{C}\right)}$. It can be shown that $U_{i}$ approximately follows a normal distribution, given by:

$$U_{i}∼N\left(\sqrt{n_{i}}\frac{p_{C}-p_{E}}{\sigma_{0}},\frac{\sigma_{A}^{2}}{\sigma_{0}^{2}}\right).$$

Knowing the distribution of $U_{i}$ under the null hypothesis and the alternative hypothesis, we can compute the Type I error rate, the power, the expected sample size, and other characteristics of a design D, and thus find an optimal adaptive design according to the procedure laid out in Section 2.2.

### 4.2 Example

In the trial by Schaub et al. [26], it was investigated whether intraoperative application of 5‐fluorouracil and low‐molecular‐weight heparin offers benefits in patients undergoing vitreo‐retinal surgery for primary rhegmatogenous retinal detachment. The primary endpoint of the trial was the occurrence of proliferative vitreoretinopathy Grade 1 or higher within 12 weeks after treatment. The trial was planned as a group‐sequential trial and with one interim analysis with equal allocation between the control arm and the experimental arm. For the planning, equal rates of $p_{C}=p_{E}=0.35$ were assumed in the control and experimental group under the null hypothesis. A reduction by one third to $p_{E}=0.233$ in the experimental group under the alternative hypothesis was assumed to be clinically relevant.

In the original design, the two‐sided significance level was set to α=0.05. To stay within the framework of Section 2 that is based on one‐sided testing, we consider the designs with a one‐sided significance level of α=0.025. The desired power in the original paper was set to 1−β=0.8. Using O'Brien‐Fleming boundaries $c_{e}=2.796$ and $c_{2,*}=1.977$, a maximal total sample size of 2×239=478 patients was obtained. Using Equation (1), we obtain $c_{2}\left(U_{1}\right)=\sqrt{2}\times 1.977-U_{1}$ as the critical value for the second‐stage‐only test statistic. No futility boundaries were considered in that trial.

We want to compare this design with an optimal adaptive two‐stage design and illustrate its characteristics. The five‐step optimization procedure reads as follows in this case:

1. Test statistic and hypotheses are formulated according to Section 4.1.
2. The objective function is chosen as the average of the expected sample size under the null and the alternative hypothesis.
3. The design should show the same Type I error rate (α≤0.025) and power (1−β≥0.8). As no futility bounds were used in the original design, we want to keep the probability to mistakenly stop early for futility very small. We chose $P_{H_{1}}\left(U_{1}<c_{f}\right)=0.005$ for numerical stability, which has practically no impact on the operating characteristics. Furthermore, we want to reach a reasonable conditional power of at least 50% to avoid a second stage without any prospect of success, even though this condition is not met by the original design.
4. We optimize the entire design, that is, all the five parameters of the five‐tuple.
5. We solve the resulting optimization problem.

A comparison between the designs is shown in Figure 3. The optimized design (solid line) shows a slightly lower expected sample size, in particular for larger effect sizes. To be more precise, the original trial has an expected sample size of 223.67 patients under the average of the null and the alternative, while the optimal design's is only 210.95. In turn, we get drastic gains of the conditional power for smaller effect sizes, as we can flexibly add more patients in the second stage. This comes at the price of an increased maximum sample size (2×279=558 [optimal] vs. 2×239=478 [original]).

**FIGURE 3:**
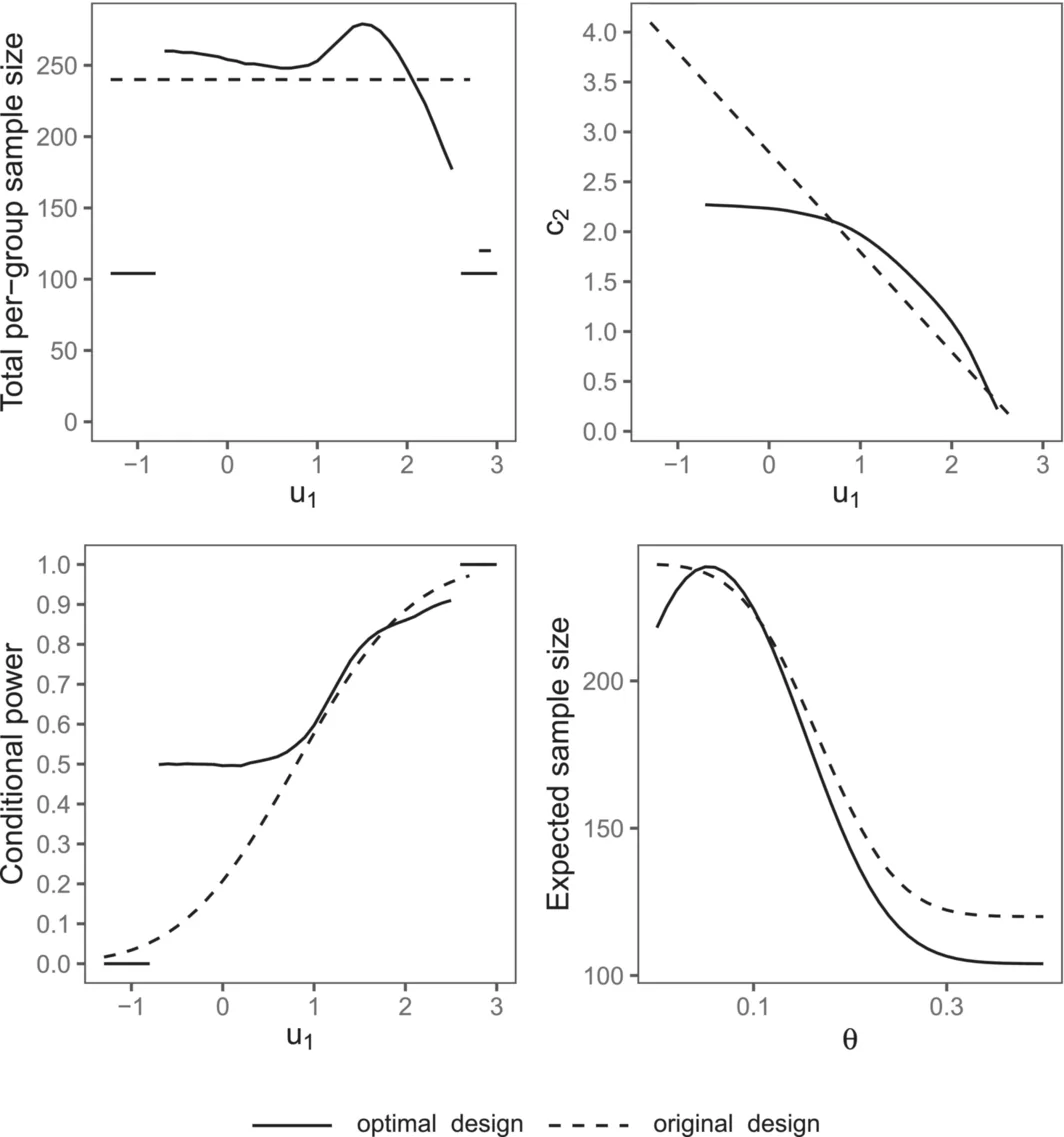
Comparison of original group‐sequential design and optimized adaptive design for the trial by [26].

## 5 Time‐To‐Event Endpoints

### 5.1 Methodology

In a trial with time‐to‐event endpoints, we typically use the log‐rank test to compare the survival distributions of two groups. Let $\lambda_{E}\left(t\right)$ be the hazard in the experimental group and $\lambda_{C}\left(t\right)$ be the hazard in the control group. Under the common assumption of proportional hazards, we denote the time‐independent hazard ratio by $\theta =\lambda_{C}\left(t\right)/\lambda_{E}\left(t\right)$. When we further assume that a lower hazard ratio is favorable, the hypotheses to test are then given by:

$$H_{0}:\theta \leq 1,\;H_{1}:\theta >1.$$

Note that we formulated the hazard ratio as the inverse of the usual convention to stay consistent with the other sections, where larger values of the test statistic indicate more evidence against the null hypothesis.

For simplicity we assume that there are no ties, so only distinct event times are observed. Let $d_{1}$ be the combined number (over both the experimental and control group) of observed events in Stage 1, and analogously, let $d_{2}$ be the number of observed events in the whole trial. Let $n_{j}^{k}$, with k∈{E,C}, be the number of patients at risk in group k just before the j‐th event. Let $1_{j}=1$ if the j‐th event was in the control group. The test statistic of the log‐rank test is then given by:

$${\mathrm{LR}}_{i}=\frac{\sum_{j=1}^{d_{i}}\left(1_{j}-\frac{n_{j}^{C}}{n_{j}^{E}+n_{j}^{C}}\right)}{\sqrt{\sum_{j=1}^{d_{i}}\frac{n_{j}^{E}n_{j}^{C}}{{\left(n_{j}^{E}+n_{j}^{C}\right)}^{2}}}},\;i\in \left\{1,2\right\},$$

and it can be shown that approximately ${\mathrm{LR}}_{i}∼N\left(\frac{1}{2}\sqrt{d_{i}}\log\left(\theta \right),1\right)$. Knowing the distribution under the null and the alternative, we can compute constraints like the Type I error rate, the power or an objective function like the expected number of events for any given five‐tuple, and thus find an optimal adaptive design according to the five‐step procedure from Figure 1.

As outlined in Section 2, we only use the stage‐wise data to compute optimal adaptive designs. This is more challenging in the survival context. In an ideal setting, we would recruit a fresh cohort for the second stage independent of the first. However, doing so would mean discarding the data of patients who survived past the interim analysis. To avoid this waste, we should reuse the patients who were recruited in Stage 1 but had not yet experienced an event at the time point of the interim analysis. This overlap breaks the independence between the populations of Stages 1 and 2, meaning the distribution of the second stage would not necessarily follow a standard normal distribution under the null hypothesis anymore.

A common solution works by using the independent increments structure, such that the statistic:

$$LR_{2}^{\prime }=\frac{\sqrt{d_{2}}{\mathrm{LR}}_{2}-\sqrt{d_{1}}{\mathrm{LR}}_{1}}{\sqrt{d_{2}-d_{1}}}$$

follows a standard normal distribution under the null and is approximately independent from ${\mathrm{LR}}_{1}$ (see [27] and for a proof [28]). This allows us to use $LR_{2}^{\prime }$ as the second‐stage test statistic.

As first mentioned in Bauer and Posch [29] and further explained by Magirr et al. [30], one has to be careful with the information that is used for adapting the trial at the interim analysis. Additional information (e.g., safety data or secondary endpoints) that correlates with survival time could be used for predicting event times. If adaptations are based on such information, this might lead to an increased Type I error rate. However, as the optimal adaptive designs considered in this manuscript only use the interim test statistic for the completely pre‐specified adaptations in number of events and second‐stage critical boundary, the aforementioned procedure (using ${\mathrm{LR}}_{1}$ and $LR_{2}^{\prime }$) is valid to control Type I error rate.

### 5.2 Example

To illustrate the application of optimal adaptive designs in the survival setting, we again consider a real clinical trial. Chen et al. [31] showed how a version of the promising zone design was applied in the context of survival trials. They used the Phase 3 trial GRAPHITE [32] as an example, which investigates whether the monoclonal antibody vedolizumab is superior to standard care in preventing patients who had undergone hematopoietic stem cell transplantation from developing aGvHD, a common and serious complication. In the following, we compare this design to an optimal adaptive two‐stage design.

The trial was a placebo‐controlled study with a 1:1 randomization ratio. The primary endpoint was intestinal aGvHD‐free survival by day 180. For the promising zone design, the necessary total number of events to achieve a power of 90% to detect a target hazard ratio of 1.704 was calculated to be 148.

The interim analysis was scheduled after 30% of the targeted primary events had been accrued, that is, after 45 events, leaving a total of 103 events to be observed in Stage 2.

The sample size reassessment was based on the CP at interim: If the CP was below 10%, the trial would be stopped for futility. In the unfavorable zone between 10% and 50%, the trial continues without adaptations, so we then conduct the final analysis after 103 events observed in Stage 2. The promising zone was set to be between 50% and 90% CP, and the sample size reassessment was defined to either increase the second‐stage number of events to 155 if CP lies between 56% and 80%, or to 129 if 50%≤CP<56% or 80%<CP≤90%. For a CP above 90%, the trial would continue without any adjustments. It is important to note that an estimation of the effect size was used for the computation of the CP instead of the assumed effect. According to Chen et al. [31], the conditional power was calculated via:

$$\mathrm{CP}=1-\Phi \left(\frac{z_{\left(1-\alpha /2\right)}-\sqrt{\frac{d_{2}}{d_{1}}}{\mathrm{LR}}_{1}}{\sqrt{1-d_{1}/d_{2}}}\right).$$

This formula can be used to translate the 10% futility boundary on the conditional power to a futility boundary for ${\mathrm{LR}}_{1}$ resulting in $c_{f}=0.491$. This corresponds to a probability of mistakenly stopping early of $P_{H_{1}}\left({\mathrm{LR}}_{1}<c_{f}\right)=0.1$.

In contrast, we illustrate what an optimal adaptive two‐stage design for this trial would look like. We use the test statistics from the log‐rank test as outlined in Section 5.1 and follow the steps from Section 2.2:

1. Test statistic and hypotheses are formulated according to Section 5.1.
2. The objective function is chosen as the average of the expected sample size under the null and the alternative hypothesis
3. The design should show the same Type I error rate (α≤0.025) and power (1−β≥0.9) as the original design. The probability to stop for futility should not exceed 0.1. The maximum number of events of 200 should not be exceeded. The original design shows high conditional power, so we require a minimum conditional power of 80%.
4. We optimize the entire design, that is, all the five parameters of the five‐tuple.
5. We solve the resulting optimization problem.

Figure 4 shows a comparison between the characteristics of the original promising zone design and an optimized version. Due to the potential futility stop, the original design does not fully meet its power requirement anymore, as it only shows a power of 84.7%. We further see that it shows a higher maximal number of events, and the slightly increased conditional power comes with the price of an increased expected number of events as can be seen in the bottom right panel. Under the alternative, the expected number of events of the promising zone design is 142, while the optimal design shows an expected number of events of only 116.

**FIGURE 4:**
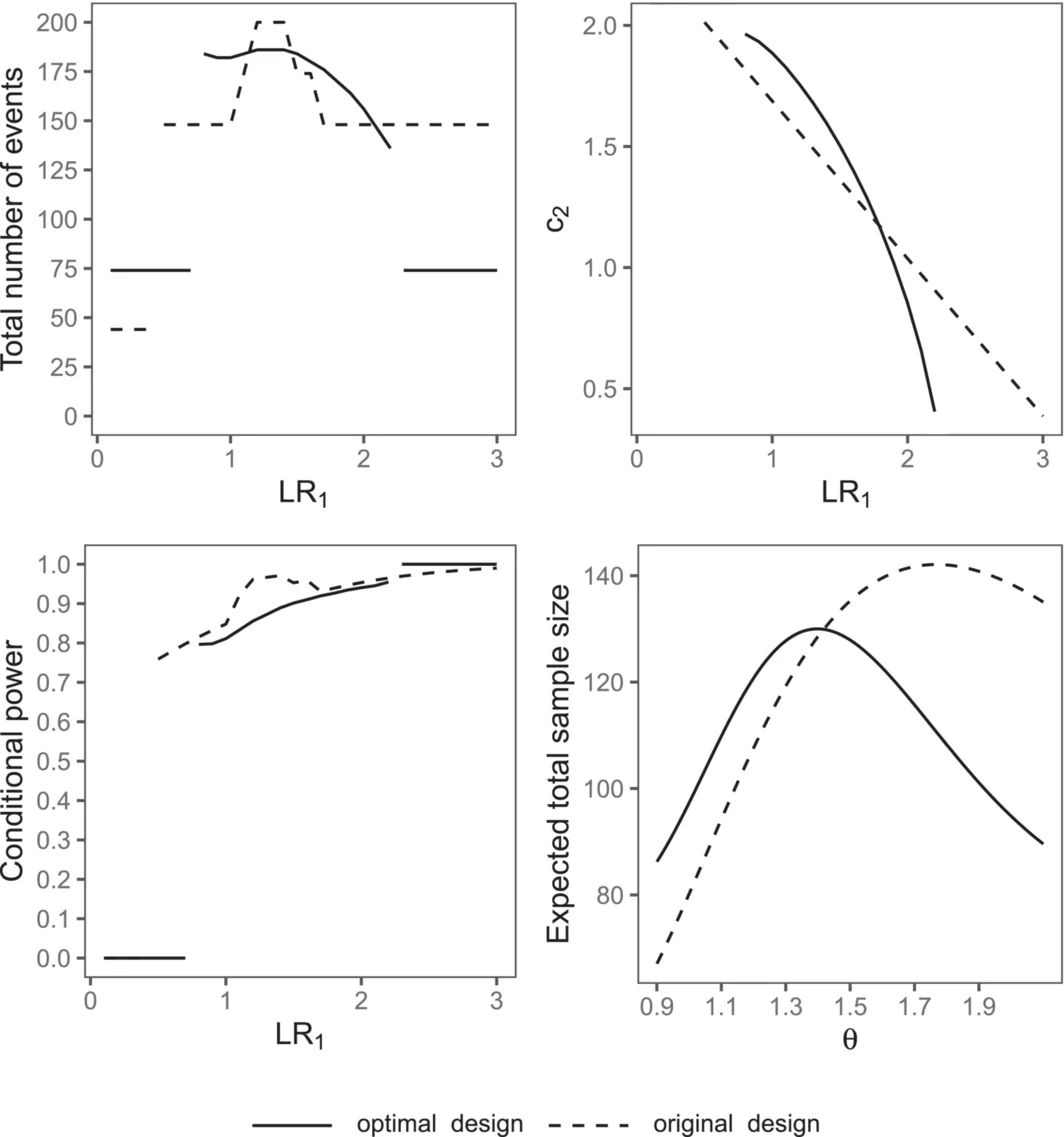
Comparison of original promising zone design and optimized adaptive design for the trial by [31].

## 6 Optimal Adaptive Designs and Two‐Sided Testing

### 6.1 Theoretical Considerations

In the previous sections, we always made the assumption of one‐sided testing by using the decision rules $X_{1}>c_{e}$ and $X_{2}>c_{2}\left(X_{1}\right)$ for rejecting the null hypothesis at the interim and the final analysis, respectively. Up to here, we therefore only compute optimal adaptive designs for hypotheses of the type:

$$H_{0}:\theta_{E}\leq \theta_{C},\;H_{1}:\theta_{E}>\theta_{C}.$$

However, in many clinical trials, two‐sided test problems are assessed.

While two‐sided testing in the context of adaptive designs was already studied in the past [25, 33], we want to highlight its pitfalls for optimal adaptive designs specifically.

When we conduct a two‐sided test, the efficacy and futility regions change. Now both large positive and large negative values of $X_{1}$ should lead to a rejection of the null hypothesis, while intermediate values lead to continuation of the trial and small absolute values to stopping for futility. This means that we now have two regions for early stop for efficacy and continuation respectively, and a single futility region in between, see Figure 5.

**FIGURE 5:**
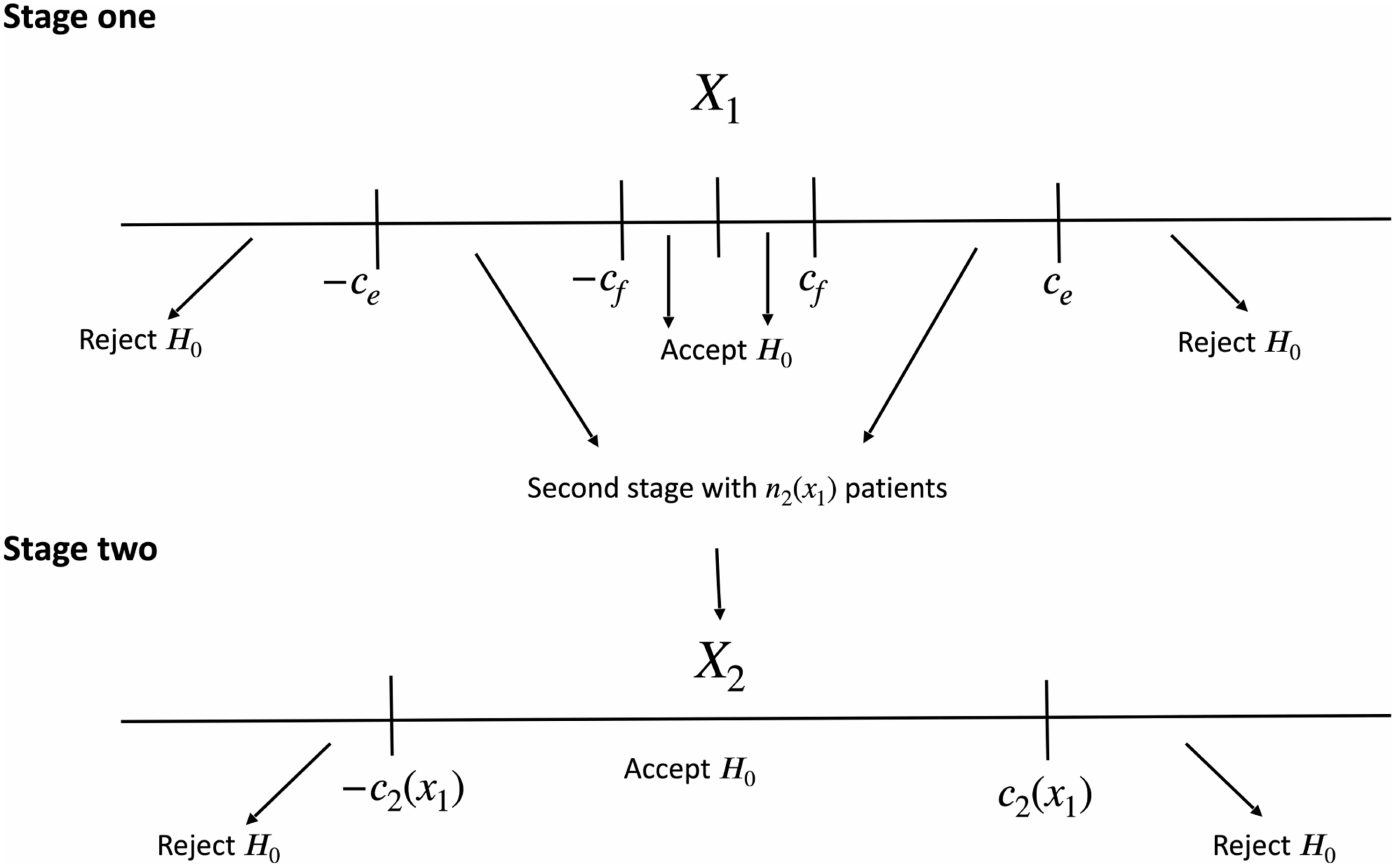
Structure of the continuation, efficacy, and futility regions in an adaptive two‐stage design with two‐sided testing.

The remaining question is how to compute an optimal adaptive two‐stage design in the two‐sided setting.

The most straightforward approach would be to compute the optimal one‐sided adaptive design $D_{\mathrm{OS}}$ at significance level α/2 and then simply mirror the decision boundaries $c_{f},c_{e}$ and $c_{2}\left(\cdot \right)$ at 0. We can decompose the Type I error of the one‐sided design via $\alpha /2=\alpha_{1,\mathrm{OS}}+\alpha_{2,\mathrm{OS}}$, where $\alpha_{1,\mathrm{OS}}$ and $\alpha_{2,\mathrm{OS}}$ denote the Type I error spent in each stage.

The Type I error of the two‐sided design with mirrored boundaries is then given as follows:

$$\begin{array}{ll} & \underset{≔\alpha_{1}}{\underbrace{P_{H_{0}}\left(X_{1}\in \left(-\infty ,-c_{e}\right)\cup (c_{e},\infty )\right)}}\\ & \;+\underset{≔\alpha_{2}}{\underbrace{P_{H_{0}}\left(\{|X_{2}|>c_{2}\left(|X_{1}|\right)\}\cap \{|X_{1}|\in (c_{f},c_{e})\}\right)}}. \end{array}$$

Assuming symmetry of $X_{1}$ and $X_{2}$ under the null hypothesis, it is immediately clear that $\alpha_{1}=2\alpha_{1,\mathrm{OS}}$. The second term can be written as:




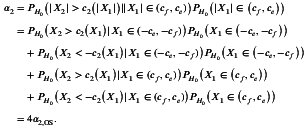




Thus, mirroring the boundaries doubles the probability of continuing to Stage 2, resulting in the second‐stage Type I error being four times as high as in the one‐sided design, which is double the intended Type I error. The essential issue lies in the fact that by mirroring the boundaries, we effectively use the test statistics $|X_{1}|$ and $|X_{2}|$ and we discard the direction of evidence. This means that we could observe $-X_{1}$ in the first stage and would reject the null if $X_{2}>c_{2}\left(-X_{1}\right)$ (or the other way round), even though this implies rejection despite contradicting evidence between the stages. This is not only counter‐intuitive and difficult to justify from a heuristic point of view, but also leads to Type I error inflation. As a consequence and in contrast to a one‐stage design, we should not simply mirror boundaries to obtain a two‐sided adaptive design from a one‐sided design.

A solution would be to only count rejections where the sign of $X_{2}$ matches the sign of $X_{1}$. We then get back the correct Type I error level. Without restrictions on $c_{f}$ and $c_{2}\left(\cdot \right)$, this would imply a certain loss of power, even though this loss of power is negligible when the sample size is sufficiently large [25]. As we typically obtain positive futility bounds and a positive $c_{2}\left(\cdot \right)$ function in optimal adaptive designs, we do not have any loss in power at all.

Of course, it is possible to compute an optimal adaptive design by the minimization procedure outlined in Section 2 using $|X_{i}|$ or, equivalently, $X_{i}^{2}$ as test statistics. We would obtain a design with correct Type I error level and the desired power. However, to ensure that the minimization algorithm fulfills the Type I error constraint, the resulting $c_{2}\left(\cdot \right)$ function takes larger values than in the one‐sided case, as it is twice as likely to continue to the second stage. This in turn implies that the sample size must be larger to achieve the same power level. See Section 6.2 for a direct comparison between such a design and the optimal one‐sided design.

A third alternative would be to use the cumulative test statistic $X_{2}^{*}$ for the second stage. This implies that the first‐stage data is used to compute the second‐stage decision as well, and observing contradictory outcomes between the stages therefore becomes negligible. While optimizing the boundaries and sample size using $X_{1}$ and $X_{2}^{*}$ would be possible in theory (the necessary calculations to determine P(Reject) would be similar to those in [34]), it would become computationally far more complex. The optimization framework in this manuscript uses stage‐wise statistics to facilitate computations and to avoid dependency structures between the stages. Therefore, optimization using the cumulative test statistic, although theoretically possible, cannot be executed by this approach and would be computationally more challenging.

Consequently, the recommended procedure is as follows: compute the optimal one‐sided design, mirror the boundaries and only reject in the second stage if the sign of the test statistic matches the sign of the first stage.

### 6.2 Illustration

We revisit the example from Section 4.2 to illustrate what happens when we optimize the design using a chi‐squared test statistic which is commonly used to test the two‐sided analogue to the test problem given in Section 4.1.

We now use a two‐sided significance level of α=0.05 and a power of 1−β=0.8. For the original study, we square the O'Brien‐Fleming boundaries $c_{e}=2.796^{2}$ and $c_{2,*}=1.977^{2}$ for the conduct of a chi‐squared test within the group‐sequential design.

Let us now compute two optimal adaptive two‐stage designs to compare them with the original group‐sequential design. For simplicity, we do not make any restrictions on conditional power or early futility stopping here and minimize the expected sample size under the alternative hypothesis.

The first design $D_{\mathrm{TS}}$ is calculated by using the stage‐wise chi‐squared test statistics, which should, following the discussion in Section 6.1, suffer from inflated sample sizes. The second design $D_{\mathrm{OS}}$ is computed by using the one‐sided test based on the normal approximation test statistics. While the trial structure of $D_{\mathrm{TS}}$ is strictly the same as outlined in Section 2, note that the evaluation of $D_{\mathrm{OS}}$ must account for the signs of the test statistics to adhere to the Type I error constraint.

The results are shown in Figure 6. For the computation of the designs, we assume that $p_{C}=0.35$. The x‐axes of the first three plots represent the outcome of the first stage in terms of the chi‐squared test statistic. As expected, we see that the $c_{2}\left(\cdot \right)$ function of the inflated optimal design yields strictly larger values than the $c_{2}\left(\cdot \right)$ function of the optimal design. As a consequence, the expected sample size of this design is also strictly larger than the expected sample size of the optimal adaptive design. The dip in the expected sample size functions, centered at p=0.35, is due to introduction of the futility bounds in the optimized designs. The expected sample size of design $D_{\mathrm{TS}}$ under the alternative is given by ${\mathrm{ESS}}_{H_{1}}\left(D_{\mathrm{TS}}\right)=211.2$, while the expected sample size of the optimal design $D_{\mathrm{OS}}$ is ${\mathrm{ESS}}_{H_{1}}\left(D_{\mathrm{OS}}\right)=193.7$.

**FIGURE 6:**
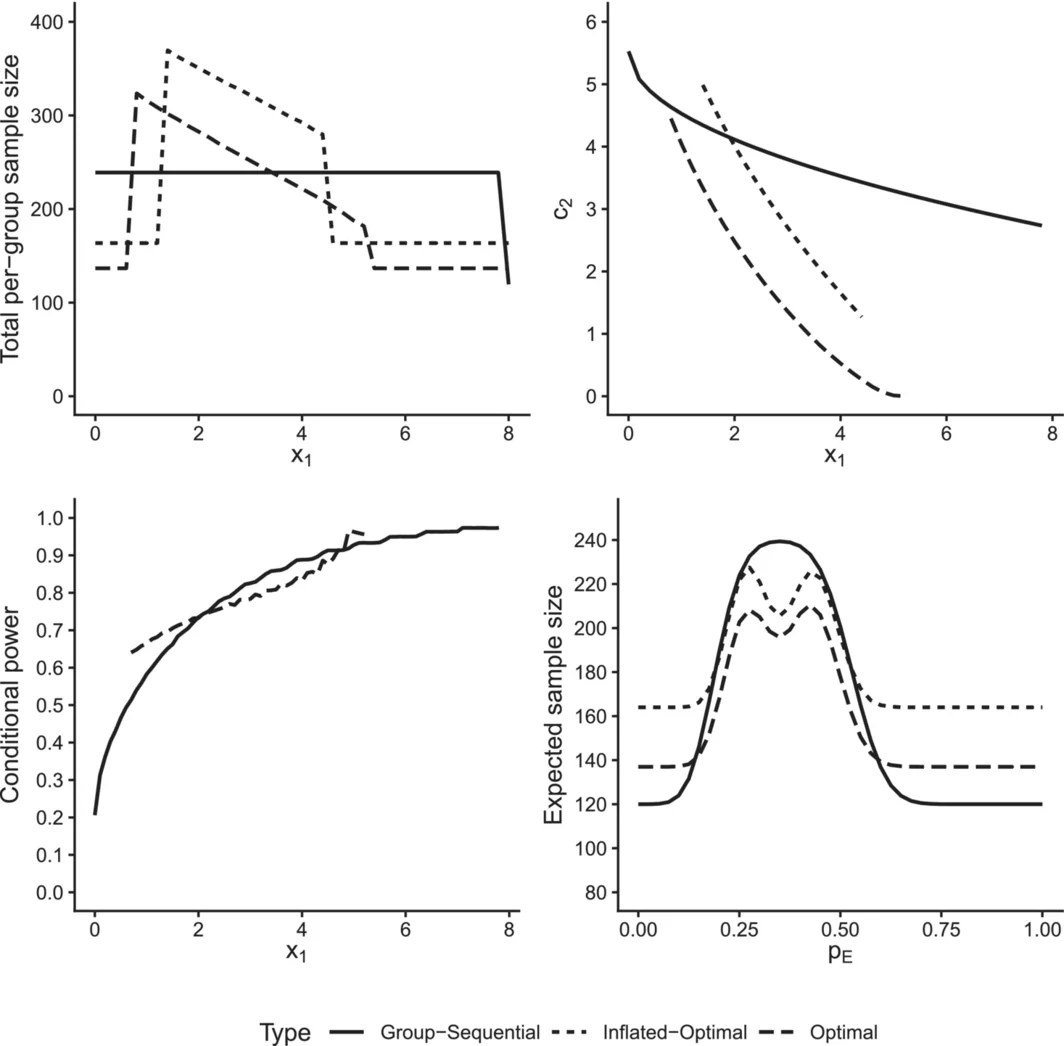
Comparison of the original group‐sequential design and the two optimized designs, where one design is optimized using the two‐sided test statistic and the other design is optimized using the one‐sided test statistic.

## 7 Remarks on Extensions to More Than Two Groups

In the previous sections, we focused on the comparison of two groups. However, in clinical research, one often encounters the situation where more than two treatments or doses are compared. In this section, we briefly discuss how multi‐arm extensions might be suitable for the optimization framework.

As described in Section 2, the designs considered throughout this paper use a single test statistic at the interim and the final analysis. One possible test problem to compare K groups via a single test statistic is given by:

$$H_{0}:\theta_{1}=\cdots =\theta_{K}\;\mathrm{vs}.\;H_{1}:\exists k,l\in \left\{1,\ldots ,K\right\}\;\text{such}\;\text{that}\;\theta_{k}\neq \theta_{l}.$$

This test problem can be addressed by an F‐test for normally distributed endpoints (Chapter 10.1 in [35]), a chi‐squared test for binary endpoints (Chapter 24.13 in [35]), or a log‐rank test extension for time‐to‐event endpoints (Chapter 2.7 in [36]). These tests all have well‐known distributions under the null and the alternative hypothesis, so we can find optimal adaptive designs for this multi‐arm setting by following the same five‐step procedure as outlined in Section 2.2. The F distribution and the chi‐squared distribution are implemented in the R package adoptr and can be used for the optimization of multi‐arm adaptive designs.

However, using this framework has limitations and might be impractical in some cases. First, the problems discussed in the previous section regarding two‐sided testing hold in this case as well. All the aforementioned tests for multiple groups are inherently two‐sided and do not provide a “direction of evidence” besides the rejection of the null hypothesis. For example, assume we compare three group means $\mu_{1},\mu_{2},\mu_{3}$ via an F‐test. A high value of ${\hat{\mu }}_{1}\gg {\hat{\mu }}_{2},{\hat{\mu }}_{3}$ could provide evidence against the null in the first stage, leading to a rather small decision boundary $c_{2}$ for Stage 2. If we then observe ${\hat{\mu }}_{2}\gg {\hat{\mu }}_{1},{\hat{\mu }}_{3}$ in the second stage, this could be sufficient evidence against the null again and we would reject it, even though the evidence per se from the two stages is conflicting.

Second, further thought should be given whether using the two‐stage framework discussed throughout this manuscript is suitable for the multi‐arm setting at all. Stopping early would mean that we either have sufficient evidence that there is a difference between the groups, or not enough evidence to continue. In the first case, we would reject the null hypothesis, but we would not know which groups differ from each other and post hoc analyses would be required. In the second case, we would not have enough evidence to continue, but some treatments might still be promising and worth being investigated in a second stage. Therefore, it is more likely that a sponsor would want to continue with a subset of the treatments instead of stopping the trial entirely.

Third, a far more common clinical trial with multiple groups is given by a design where multiple treatments are compared to a single control, but not to each other. Such designs can be seen as a simple version of platform trials [37]. The framework then drastically shifts to a testing problem with multiple null hypotheses $H_{1},\ldots ,H_{K}$. Such a testing problem is typically addressed by closed testing procedures, where a null hypothesis $H_{i}$ is only rejected if all of the intersection hypotheses $H_{ℐ}≔\cap_{j\in ℐ}H_{j}$ are rejected, where ℐ∈{J|J⊆{1,…,K},i∈J}. There is extensive literature on this topic (see [38, 39, 40]), also regarding its use in the context of adaptive designs. These concepts can be further extended to not only include multiple arms, but multiple endpoints as well. To facilitate the construction of such multiple testing procedures, graph‐based methods were proposed [41] and afterwards extended to adaptive designs (see [42, 43]).

While there exists some literature on optimal multi‐arm multi‐stage (MAMS) designs with a single primary endpoint [44, 45], examining potential optimal adaptive designs for these kinds of testing problems is beyond the scope of this paper and a topic for future research.

## 8 Discussion

In this article, we showed that optimal adaptive designs can be effectively used in a variety of clinical scenarios beyond normally distributed endpoints. We gave guidance on how the optimization procedure can be applied in practice. We further compared the characteristics of optimal adaptive designs to study designs that were planned and used in real clinical trials. We showed that, while group‐sequential designs are easy to implement and understand, a substantial gain in efficiency may be achieved by optimizing the parameters. In a subsequent section, we discussed the relevance of two‐sided testing. While having been studied in the past, we thoroughly explained why one should not simply mirror optimal one‐sided boundaries without matching the signs of the stage‐wise statistics. Additionally, we showed why one should not optimize the design's features using a two‐sided test statistic directly. It is possible in theory, but we illustrated that this leads to suboptimal designs with inflated sample sizes and decisions that are difficult to interpret. The last section was devoted to the discussion of multi‐arm extensions. While it is possible to use the same optimization framework for the testing problem of multiple groups, we highlighted that this might not always be suitable in practice. The issue of conflicting evidence between the stages persists, and the interpretation of early stopping for efficacy or futility is more difficult.

The optimization of adaptive designs becomes arguably more critical in the context of rare diseases or small populations, where patient recruitment is challenging. In these settings, the ability to minimize the necessary number of patients or events while keeping a high power level is of great importance. In this paper, we showed and illustrated that the general procedure is applicable to a wide range of endpoints and trial settings. However, we still rely on the asymptotic behavior of the test statistics, which may break in ultra‐rare diseases with very limited sample sizes. For normally distributed outcomes, Kieser et al. [17] showed that it is possible to stay within this optimization framework while using the exact distribution of the test statistic.

While we only considered the minimization of the expected sample size or number of events in this paper, the optimization framework is flexible and allows for other objectives as well. For instance, Held et al. [46] proposed the so‐called “Experimental Unit Information Index” in a recent work that combines power, Type I error rate, and expected sample size into a single measure. More refined objectives, as higher moments of the sample size function, are discussed in [11]. Future work could investigate the design characteristics of optimal adaptive designs when optimizing such objective functions instead.

In addition, the optimization framework researched so far does not yet allow for more complex adaptive adjustments. There is a lot of current research on platform trials and basket trials, where multiple treatments and/or multiple arms are investigated within a single trial, see for example [37, 47]. While being computationally expensive, extending the current optimization framework to at least more than two stages could be a first step towards this direction.

## Funding

This work was supported by Innovative Health Initiative (101165912).

## Ethics Statement

Not applicable. This study did not involve experimental research on humans or animals.

## Consent

The authors have nothing to report.

## Conflicts of Interest

The authors declare no conflicts of interest.

## Supporting information


**Data S1:** Supplementary Code.

## Data Availability

There are no data associated with this manuscript. The R code to reproduce the results is available in the Supporting Information.
